# Distinction of *Plasmodium falciparum *recrudescence and re-infection by MSP2 genotyping: A caution about unstandardized classification criteria

**DOI:** 10.1186/1475-2875-7-185

**Published:** 2008-09-23

**Authors:** Petrica Rouse, Mtawa AP Mkulama, Philip E Thuma, Sungano Mharakurwa

**Affiliations:** 1 Johns Hopkins Bloomberg School of Public Health, Baltimore, MD, USA; 2 The Malaria Institute at Macha, Choma, Zambia

## Abstract

**Background:**

*Plasmodium falciparum *genotyping with molecular polymorphic markers is widely employed to distinguish recrudescence from re-infection in antimalarial drug efficacy monitoring programmes. However, limitations occur on agarose gel DNA measurements used to resolve the polymorphisms. Without empirical data, the current distinction of pre- and post-treatment bands, as persistent or new infection, is subjective and often varying by author. This study measures empirical tolerance limits for classifying different-sized bands as same or different alleles during MSP2 genotyping.

**Methods:**

*P. falciparum *field samples from 161 volunteers were genotyped by nested PCR using polymorphic MSP2 family-specific primers. Data were analysed to determine variability of band size measurements between identical MSP2 alleles randomized into different agarose lanes.

**Results:**

The mean (95% CI) paired difference in band size between identical alleles was 9.8 bp (1.48 – 18.16 bp, p = 0.022) for 3D7/IC and 2.54 (-3.04 – 8.05 bp, p = 0.362) for FC27. Based on these findings, pre- and post-treatment samples with 3D7/IC alleles showing less than 18 bp difference corresponded to recrudescence, with 95% confidence, while greater difference indicated new infection. FC27 allele differences were much narrower. For both 3D7/IC and FC27 amplicon, allele detection sensitivity was significantly higher with 13 μl compared to 20 μl or 30 μl lane loading volumes.

**Conclusion:**

During MSP genotyping, it is useful to standardize classifications against measurement of background variability on identical alleles, in order to obtain reliable findings. It is critical to use a fixed optimal lane loading volume for constant allele patency, to avoid the disappearance or false appearance of new infection.

## Background

Increasing resistance to antimalarial therapy is one of the major obstacles for malaria control worldwide [1-3]. Consequently, surveillance of therapeutic efficacy of these drugs over time is an essential component of malaria control.

In endemic areas, the reappearance of asexual erythrocytic stages of *P. falciparum *after a correct therapeutic course may be attributed to either recrudescence of drug-resistant parasites or a new infection. Molecular genotyping with MSP1, MSP2 and GLURP polymorphic markers is now widely used to distinguish recrudescence from re-infection during *in vivo *drug efficacy monitoring programmes[4,5].

Infections can be distinguished by polymerase chain reaction (PCR) fingerprinting. PCR has important potential advantages for being highly sensitive and specific in detecting malaria parasitaemia[6]. Comparing pre- and post-therapy isolates provides information about the clonality of any persistent infection. However, although straightforward in principle, the current methods used to interpret results vary widely[7]. Band size variability between same alleles can occur on agarose gels. Current classifications of alleles as same or different are often subjective and varying between authors, with no empirical data. A recent MMV/WHO consensus report on *P. falciparum *genotyping[8] recommends possible use of polyacrylamide gels, capillary electrophoresis, or use of dedicated fragment sizing software, to increase discriminatory power. However, band size variability between same alleles can still occur, leading to false categorization as different alleles. The present study examines band size variance within and between MSP2 alleles and measures empirical thresholds for classing infections as same or different.

## Methods

### Study area and data collection

The study was conducted in a 2000 km^2 ^area from the vicinity of the Malaria Institute at Macha, in Southern Province of Zambia. The area encompasses four chiefdoms (*Mapanza*, *Chikanta*, *Macha *and *Muchila*), with an estimated population of 180,000 and experiences hyperendemic malaria transmission. The study was based on a simple random sample of 161 thick film-positive residents of all ages and either sex. Volunteers who gathered at designated central areas were screened for malaria by microscopy. *P. falciparum *DNA samples were collected by blotting fingerprick blood from thick film-positives onto Whatman No 3 MM filter paper as previously described [9].

Informed consent was sought from each participant and in the case of children, from their guardians. Ethical clearance was obtained from both the national and John Hopkins University IRB's.

### DNA extraction and PCR

*P. falciparum *DNA was extracted from air-dried filter paper blood samples using the chelex method[10,11]. The extracted DNA was amplified using nested PCR and MSP2 family specific primers as previously described[12]. First-round PCR primers corresponded to conserved sequences flanking these regions. Second-round PCR primers were used to amplify the 3D7/IC and FC27 allelic families of MSP2[12]. For both primary and secondary reactions a 100 μl total volume was used comprising: 8 μl template, 0.25 μM MSP2 primers, 1.5 mM magnesium chloride, 200 μM dNTP's, 1× PCR Buffer and 4 U of Taq DNA polymerase. PCR annealing cycles for both primary and secondary MSP2 reactions consisted of an initial two minute denaturation step at 94°C, followed by 25 cycles of denaturation at 94°C for 45 seconds, annealing at 61.1°C for 45 seconds and extension at 65°C for one minute[9]. Final extension was at 65°C for two minutes. The PCR amplifications were performed in a Thermo Electron^® ^PX2 (HBPX2) thermal cycler.

### Agarose gel electrophoresis

PCR product was resolved by electrophoresis on ethidium bromide-stained 1.5% agarose gels, on a standard 11 cm × 9 cm casting tray. Gel thickness was 1.5 cm with well sizes of 1.2 cm depth × 0.7 cm length × 0.1 cm width. Duplicate amplicon for each sample was individually randomized and loaded separately in 13 ul volumes per lane (denoted lane A and lane B). Subsequently, each sample amplicon was also loaded into randomized single lanes in 20 ul and then 30 ul volumes. Therefore, because of randomization, some duplicate samples were run in adjacent or non-adjacent lanes. PCR products for 3D7/IC and FC27 family specific primers were run in separate lanes and bands were visualized by UV transillumination. Molecular weight, band mass and intensity were measured using the Kodak 1D Imaging system.

### Statistical analysis

Data were analysed to determine the mean and 95% confidence limits of paired differences in band size between identical MSP2 alleles randomized into different agarose lanes. Linear regression was used to study the relationship between paired band size mean difference and allele size after controlling for confounders. Potential confounders included locus used, participants' age and sex, allele intensity, allele mass and streak pattern. Paired-sample t test was used to determine if means statistically differed by volume and locus. McNemar's test was used to calculate MSP2 allele patency by lane loading volume and Wilcoxon Z test used to compare number of alleles detected.

## Results

One hundred and sixty one samples were used to assess the tolerance limits for band size classifications. All genotyping was done using MSP2 with varied volumes of 13 ul, 20 ul and 30 ul. Participants were aged between 2 – 61 years (median 10 years) with an even distribution between the sexes. Asexual parasite density ranged from 0 – 16560 parasites/μl, with a geometric mean (95% CI) of 364 (119 – 610) asexual parasites/μl.

### Inter-lane variability in 3D7/IC and FC27 allele size measurements

**Figure 1:**
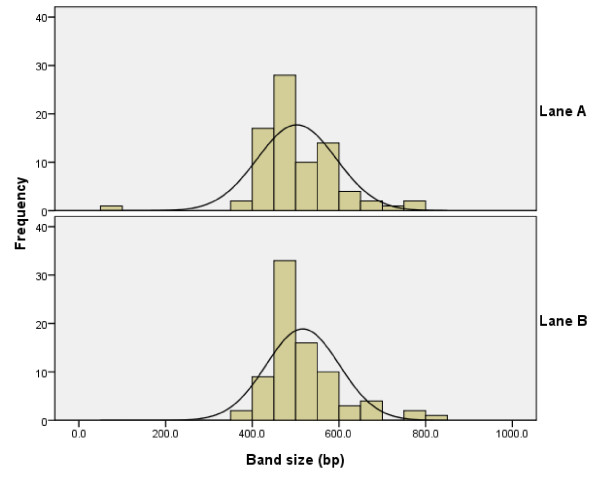
**Frequency distribution of 3D7/IC allele sizes (bp)**. Lane A: based on readings from sample amplicon duplicate randomized into lane A (13 μl loading volume). Lane B: based on readings from sample amplicon duplicate randomized into lane B (13 μl loading volume).

Despite carrying duplicates of the same amplicon, lane A and lane B exhibited different band size measurements for 3D7/IC alleles (Figure 1). Mean (95% CI) 3D7/IC allele sizes were 501.8 bp (481.4 – 522.1 bp) and 516.5 bp (497.7 bp – 535.3 bp), for the same group of samples in lane A and lane B, respectively. The paired difference in band size between identical 3D7/IC amplicon run in lane A and lane B was 9.8 bp (95% CI: 1.48 – 18.16 bp; p = 0.022). Thus band sizes for identical 3D7/IC alleles differed by up to 18 base pairs on agarose gels. The mean band intensity and number of streaked lanes were not statistically different for lane A and B. The respective mean intensities (95%CI) of lanes A and B were (135 – 156) pixels and 139 (128 – 149) pixels with a difference of 7 (-5 – 19) pixels.

### Inter-lane variability in FC27 allele size measurements

**Figure 2:**
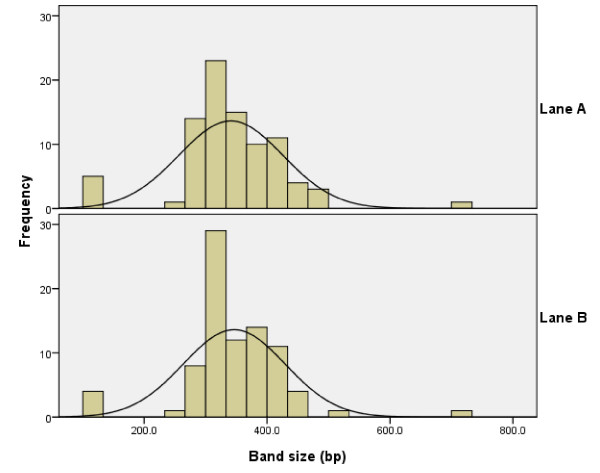
**Frequency distribution of FC27 allele sizes (bp)**. Lane A: based on readings from sample amplicon duplicate randomized into lane A (13 μl loading volume). Lane B: based on readings from sample amplicon duplicate randomized into lane B (13 μl loading volume).

The mean (95% CI) FC27 allele sizes were 341.0 bp (322.9 – 359.1 bp) and 346.4 bp (328.5 – 364.3 bp), respectively for the same samples ran in lane A and lane B. While the mean (95% CI) paired difference in band size between identical FC27 alleles was 2.5 bp (-3.97 bp – 8.05 bp; p > 0.362). Although carrying duplicates of the same amplicon, lane A and lane B exhibited different band size measurements for FC27 alleles (Figure 2).

### MSP2 allele detection in relation to gel loading volume

There were no significant differences in detected allele size distribution by sample loading volume. Using 13 ul, the mean (95% CI) 3D7/IC allele sizes were 502.6 bp (95% CI: 482.4 – 522.7 bp) in lane A; 516.5 bp (95% CI: 497.7 – 535.3 bp) in lane B; 487.3 bp (95% CI: 457.6 – 516.9 bp) in 20 ul lane; and 479.3 bp (95% CI: 448.7 – 509.9 bp) in 30 ul lane (p = 0.182).

Corresponding mean (95% CI) readings for FC27 were 341.0 bp (95%CI: 322.9 – 359.1 bp) in 13 ul lane A; 346.4 bp (95% CI: 328.5 – 364.3 bp) in 13 ul lane B; 345.4 bp (95% CI: 325.4 – 365.4 bp) in 20 ul lane and 350.1 bp (333.1 – 367.2 bp) in 30 ul lane (p = 0.921).

However, for both 3D7/IC and FC27 loci, the mean number of alleles per sample and the total number of amplified alleles detected were higher with 13 ul than 20 ul or 30 ul lane loading volumes (Table 1 – Table 2).

**Table 1:** Allele detection per sample by loading volume and MSP2 locus.

**Lane loading volume**	**Mean number of alleles per sample [range]**			
	
	**3D7**	**ψ Wilcoxon Z (P value)**	**FC27**	**ψ Wilcoxon Z (P value)**
13 μl (A)	1.54 [0–4]	-	1.45 [0–6]	-
13 μl (B)	1.54 [0–3]	0.000 (1.000)	1.44 [0–5]	-0.577 (0.564)
20 μl	1.53 [1-4]	-1.890 (0.059)	1.22 [0–3]	-2.801 (0.005)
30 μl	1.49 [0–4]	-2.333 (0.020)	1.12 [0–3]	-3.788 (< 0.001)

ψ Paired differences tested against 13 ul lane A.

**Table 2:** MSP2 allele patency by lane loading volume.

**MSP2 Locus**	**Lane loading volume**						
	
		**13 ul (B)**		**20 ul**		**30 ul**	
3D7/IC	13 μl (A)	+	-	+	-	+	-
	+	79	1	74	6	73	7
	-	1	12	1	1	0	2

* P		1.000		0.125		0.016	

FC27	13 μl (A)	+	-	+	-	+	-
	+	84	2	71	14	65	21
	-	1	1	1	0	1	0

* P		1.000		0.001		< 0.001	

* McNemar's test run for 13 μl lane (A) compared to other lanes. For both 3D7/IC and FC27, the number of alleles detected was greatest when 13 μl loading volume was used

### Regression model

The variability seen in paired band size mean difference was not accounted for by sex, age, allele intensity, allele mass or locus used.

## Discussion

In antimalarial drug efficacy monitoring programmes, molecular genotyping with MSP1, MSP2 and GLURP polymorphic markers is widely used to distinguish recrudescence from re-infection. However, one of the current limitations for this invaluable "PCR correction" is that similar-sized alleles are often classed as same or different infections, using subjective visual inspection on agarose gels. Other workers have adopted a 10 bp cut off point [5] illustrating arbitrary variability between authors, with no standardizing experimental data.

The present study ascertained tolerance limits for empirically classing different-sized bands as recrudescence or re-infection, during MSP2 genotyping. This classification is the key basis for identifying drug therapeutic failure from new infections during *in vivo *drug efficacy assessments. The results show that band size measurements for the same allele can vary appreciably on agarose gels, presumably for varied reasons, ranging from gel running conditions, human error and complexity of infection. Identical MSP2 alleles could differ in band size, by up to 18 bp for 3D7/IC and 11 bp, for FC27 families. Therefore, visual distinction of infections is not reliable. For the reported data, post-treatment alleles exhibiting differences below these upper limits could thus be classed as recrudescence, with 95% confidence, while those above these thresholds could be considered as re-infection.

Neither band size nor lane streaking was significantly different by loading volume. Mean allele size was significantly larger for 3D7/IC than FC27 and not dependent on participants' age or sex, allele intensity or mass. Readings from a 13 ul loading volume detected significantly more 3D7/IC and FC27 alleles than higher 20 ul and 30 ul volumes.

The current study estimated absolute band size variability for identical alleles randomized on agarose gel lanes. During routine genotyping for drug efficacy monitoring trials, pre and post samples are generally placed in adjacent wells when viewing. This may lead to narrower allele differences than reported. Furthermore, the measurements cannot be generalized for different laboratories and separation systems. Individual laboratories would need to measure within allele variability for their own electrophoresis apparatus to standardize their genotyping classifications.

## Conclusion

This study shows that it is useful to standardize MSP2 genotyping classifications against measurement of background band size variability on identical alleles, in order to obtain reliable findings. Furthermore, a fixed optimal lane loading volume is critical for constant allele patency, to avoid the disappearance or false appearance of new infection.

## Competing interests

The authors declare that they have no competing interests.

## Authors' contributions

PR implemented the algorithms and carried out the experiment including: PCR genotyping, data collection, data entry, data analysis and manuscript preparation. MAPM assisted with data entry, data cleaning and manuscript revision. PT contributed scientific critique, project support and assisted with manuscript review. SM was the project leader who developed the theory, aided with statistical analysis and preparation of the manuscript. All authors read and gave final approval of the submitted manuscript.
